# Strategies for Enhancing Participant Recruitment and Retention in Dementia Research in Africa: Experience from the African Dementia Consortium

**DOI:** 10.1002/alz70860_096562

**Published:** 2025-12-23

**Authors:** Rufus O. Akinyemi, Olufisayo Oluyinka Elugbadebo

**Affiliations:** ^1^ Africa Dementia Consortium, Ibadan, Ibadan, Nigeria; ^2^ University College Hospital, Ibadan, Oyo State, Nigeria

## Abstract

**Background:**

Achieving greater diversity and inclusion in global dementia research requires the inclusion of underrepresented geographic, ethnic and regional populations such as indigenous Africans. The African Dementia Consortium operates across multiple African countries through research projects involving interdisciplinary African researchers working across multiple research projects.

**Method:**

The Recruitment and Retention for Alzheimer's Disease Diversity Genetic Cohorts in the ADSP (READD‐ADSP) is a case/control study that is recruiting, retaining and evaluating 13,000 participants of diverse race/ethnicity, including 5,000 indigenous Africans (AF), 4,000 Black Americans (BA), and 4,000 US Hispanic/Latinx (HL). The study in Africa involves multiple sites in 10 African countries operating under the African Dementia Consortium (AfDC). Barriers and facilitators of recruitment were observed and innovative interventions developed.

**Result:**

A total of 2457 participants (1251 AD cases and 1246 controls) have been recruited into the READD – ADSP Africa programme between 22nd June, 2023 and 14th January, 2025 from diverse populations in 10 countries in eastern, central and western Africa. Identified barriers to recruitment and retention include non‐existence or poorly managed dementia registries, language diversity, myths around blood donation for research, family dynamics, stigma and unmet expectations regarding incentives for participating in research and concerns about data privacy. These challenges were mitigated through innovative, context ‐ sensitive community – engaging implementation science approaches involving community advisory boards, leveraging previous research programmes, creation of novel applied theater, and use of electronic and social media.

**Conclusion:**

The collaboration of the ADSP with the African Dementia Consortium (AfDC) to recruit and retain participants in the READD – ADSP program is yielding positive results with sustained recruitment and retention of study participants.

